# Automated assessment of echocardiograms to aid diagnosis of heart failure with preserved ejection fraction

**DOI:** 10.1093/ehjdh/ztag142

**Published:** 2026-09-04

**Authors:** Constantijn Sebastiaan Venema, Max Venner, Anouk Achten, Wouter Ouwerkerk, Anne Raafs, Sanne Mourmans, Maurits Sikking, Hans-Peter Brunner-La Rocca, Stephane Heymans, Vanessa P M van Empel, Yoran Hummel, Justin Ezekowitz, Elke Hoendermis, Adriaan A Voors, Joanna J Wykrzykowska, Christian Knackstedt, Jasper Tromp, Jerremy Weerts

**Affiliations:** Department of Cardiology and Cardiothoracic Surgery, Heart Centre, University of Groningen, University Medical Centre Groningen, Hanzeplein 1, 9713 GZ Groningen, The Netherlands; Department of Cardiology, Cardiovascular Research Institute Maastricht (CARIM), Maastricht University Medical Centre (MUMC+), Maastricht, The Netherlands; Department of Cardiology, Cardiovascular Research Institute Maastricht (CARIM), Maastricht University Medical Centre (MUMC+), Maastricht, The Netherlands; Department of Dermatology, University of Amsterdam Medical Centre, Amsterdam, Netherlands; National Heart Centre, 5 Hospital Drive, Singapore 169609, Singapore; Department of Cardiology, Cardiovascular Research Institute Maastricht (CARIM), Maastricht University Medical Centre (MUMC+), Maastricht, The Netherlands; Department of Cardiology, Catharina Hospital, Eindhoven, The Netherlands; Department of Cardiology, Cardiovascular Research Institute Maastricht (CARIM), Maastricht University Medical Centre (MUMC+), Maastricht, The Netherlands; Department of Cardiology, Cardiovascular Research Institute Maastricht (CARIM), Maastricht University Medical Centre (MUMC+), Maastricht, The Netherlands; Department of Cardiology, Cardiovascular Research Institute Maastricht (CARIM), Maastricht University Medical Centre (MUMC+), Maastricht, The Netherlands; Department of Cardiology, Cardiovascular Research Institute Maastricht (CARIM), Maastricht University Medical Centre (MUMC+), Maastricht, The Netherlands; Department of Cardiology, Cardiovascular Research Institute Maastricht (CARIM), Maastricht University Medical Centre (MUMC+), Maastricht, The Netherlands; Us2.ai, 72 Circular Road, #03-01, Singapore 049426, Singapore; University of Alberta, 116 St and 85 Ave, Edmonton, Alberta T6G 2R3, Canada; Department of Cardiology and Cardiothoracic Surgery, Heart Centre, University of Groningen, University Medical Centre Groningen, Hanzeplein 1, 9713 GZ Groningen, The Netherlands; Department of Cardiology and Cardiothoracic Surgery, Heart Centre, University of Groningen, University Medical Centre Groningen, Hanzeplein 1, 9713 GZ Groningen, The Netherlands; Department of Cardiology and Cardiothoracic Surgery, Heart Centre, University of Groningen, University Medical Centre Groningen, Hanzeplein 1, 9713 GZ Groningen, The Netherlands; Department of Cardiology, Cardiovascular Research Institute Maastricht (CARIM), Maastricht University Medical Centre (MUMC+), Maastricht, The Netherlands; Department of Cardiology and Cardiothoracic Surgery, Heart Centre, University of Groningen, University Medical Centre Groningen, Hanzeplein 1, 9713 GZ Groningen, The Netherlands; National Heart Centre, 5 Hospital Drive, Singapore 169609, Singapore; Saw Swee Hock School of Public Health, National University of Singapore, and the National University Health System, Tahir Foundation Building, 12 Science Drive 2, #10-01, Singapore 117549, Singapore; Department of Cardiology, Cardiovascular Research Institute Maastricht (CARIM), Maastricht University Medical Centre (MUMC+), Maastricht, The Netherlands

**Keywords:** Heart failure with preserved ejection fraction ● Diagnosis ● Artificial intelligence ● Echocardiography ● HFpEF ● eHealth

## Abstract

**Aims:**

Diagnosis of heart failure with preserved ejection fraction (HFpEF) using the HFA-PEFF and H_2_FPEF scores remains challenging in clinical practice and relies on echocardiographic assessment. We aimed to determine whether diagnostic scoring based on automated deep learning interpretation of echocardiograms performs similar to manual measurements in diagnosing HFpEF.

**Methods and results:**

We analysed echocardiograms using an automated deep learning algorithm and manually in three cohorts: a test cohort (102 HFpEF patients diagnosed by right heart catheterization and echocardiography), an ambulatory validation cohort (129 HFpEF patients), and a diagnostic validation cohort (*n* = 427, of which 182 HFpEF and 245 non-HFpEF patients). We evaluated correlations between automated and manual HFA-PEFF and H_2_FPEF scores across cohorts, their correlation with pulmonary capillary wedge pressures (PCWP), and compared diagnostic accuracy using the area under the curve (AUC). Automated and manual measurements showed good agreement across cohorts, with good correlations between HFA-PEFF (0.78–0.86) and H_2_FPEF (0.96–0.98) scores and similar correlations with PCWP. One in five patients with high-likelihood HFpEF based on manual HFA-PEFF scores was classified as intermediate-likelihood by automated scores due to lower estimated left atrial volumes, without consistent interaction with atrial fibrillation. Areas under the curve for automated HFA-PEFF and H_2_FPEF scores did not consistently differ from manual scores {0.70 [95% confidence interval (CI): 0.66–0.74] vs. 0.71 [95% CI: 0.66–0.75] and 0.78 [95% CI: 0.73–0.82] vs. 0.75 [95% CI: 0.71–0.80], respectively}.

**Conclusion:**

The HFA-PEFF and H_2_FPEF scores based on automated and manual echocardiographic analysis showed similar diagnostic accuracy, suggesting automated HFpEF diagnosis using deep learning analysis of echocardiograms is feasible.

## Graphical Abstract

**Graphical Abstract:**
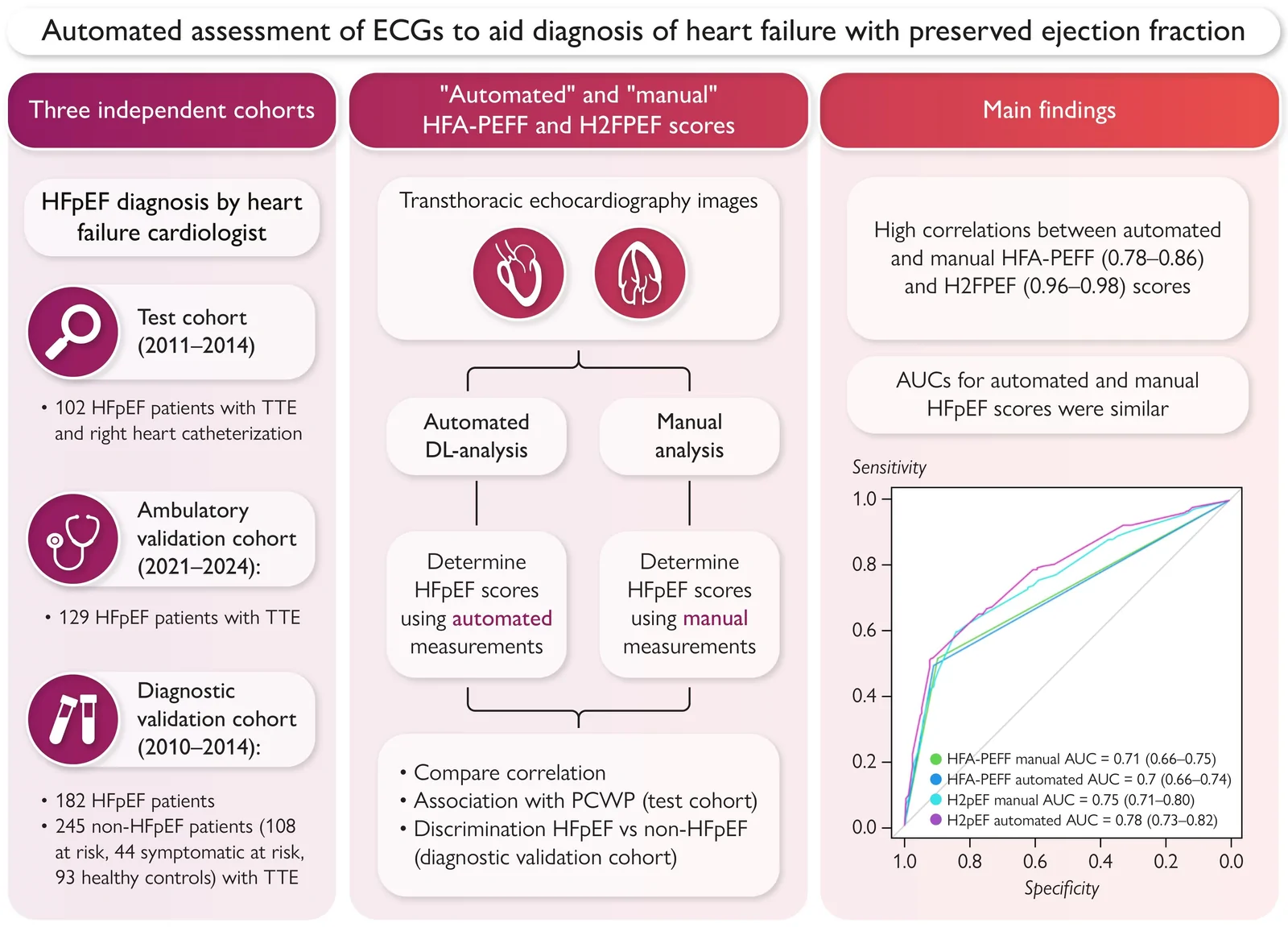

## Background

Establishing the diagnosis of heart failure with preserved ejection fraction (HFpEF) remains challenging.^1^ To aid the diagnosis in a patient with suspected HFpEF, two multiparametric scoring systems (the HFA-PEFF and H_2_FPEF scores) have been developed to determine the likelihood of HFpEF.^2–6^ Both scores include echocardiographic measurements, but these measurements are time-consuming and subject to interobserver variability.^7–10^ It has been demonstrated that automated deep learning evaluation of echocardiographic images is associated with reduced measurement variability and has been validated in patients with heart failure.^9,11^ Implementing automated echocardiographic measurements may therefore facilitate the application of diagnostic HFpEF scores in clinical practice by saving time and resources.

## Aims

We hypothesized that using automated echocardiographic measurements to determine HFpEF scores yields similar diagnostic accuracy as manual measurements. Thus, we aimed to determine whether diagnostic HFpEF scoring based on fully automated deep learning interpretation of echocardiographic images is accurate and feasible compared with manual interpretation.

## Methods

### Study design

For this study, we used data from three cohorts: a test, an ambulatory, and a diagnostic validation cohort. For each cohort, all HFpEF diagnoses were established as a clinical diagnosis by heart failure cardiologists according to the leading European Society of Cardiology HF guidelines.^1^ All participants provided written informed consent, and all cohorts were approved by local Ethics Review Committees.

### Cohorts

The test cohort consisted of 102 patients with symptomatic HFpEF who were included in a randomized trial investigating the effect of sildenafil on pulmonary artery pressure between October 2011 and September 2014 (ClinicalTrials.gov, NCT01726049).^12^ Patients underwent right heart catheterization with measurement of pulmonary capillary wedge pressure (PCWP) and simultaneous transthoracic echocardiography. Heart failure with preserved ejection fraction was defined as signs and symptoms of HF [New York Heart Association (NYHA) functional class II-IV], despite HF therapy, and a left ventricular ejection fraction (LVEF) ≥ 45%, mean pulmonary artery pressure (PAP) > 25 mmHg, and mean PCWP > 15 mmHg.

The ambulatory validation cohort comprised 129 patients with HFpEF from a dedicated HFpEF outpatient clinic between October 2021 and May 2024 with all evaluated echocardiographic parameters available by both methods (ClinicalTrials.gov, NCT04976348).^13^ The diagnostic workflow has been detailed elsewhere.^14^

Finally, the diagnostic validation cohort was derived from the Alberta Heart Failure Etiology and Analysis Research Team (HEART) study and consisted of 182 patients with HFpEF and 245 patients without HFpEF, of which 108 patients at risk of HFpEF, 44 patients at risk of HFpEF with symptoms of other diseases, and 93 age- and sex-matched healthy controls (Alberta Heart Failure Etiology and Analysis Research Team; ClinicalTrials.gov, NCT02052804).^15^ Participants were prospectively recruited between 2010 and 2014 and adjudicated into each group by two blinded investigators.

### Echocardiographic analysis and calculation of diagnostic scores

Certified sonographers manually analysed all echocardiograms from the three cohorts according to local standards. In addition, all echocardiograms were processed using an automated, validated deep learning workflow (Us2.ai, version 2.2, Singapore). These algorithms automatically identify the correct 2D or Doppler view, annotate and segment structures, and provide echocardiographic measurements.^11^

Subsequently, the HFA-PEFF and H_2_FPEF scores were determined in all cohorts using manual echocardiographic measurements (further referred to as manual scores) and deep learning-derived measurements (further referred to as automated scores). In case of missing data in the score components, data were imputed five times using Multiple Imputation by Chained Equations with predictive mean matching.^16^ All echocardiographic, demographic, and medical history variables were used as predictors for imputation. Derived variables such as ratios were computed after imputation. The scores were then averaged over the imputed datasets.

### Statistical analysis

The yield of manual and automated echocardiographic measurements was determined in each cohort, and correlations were assessed using Pearson’s correlation coefficient. The differences between manual and automated measurements were quantified using the root mean square error (RMSE) and using Bland–Altman bias. The differences in classification between manual and automated HFA-PEFF and H_2_FPEF scores were visualized using Sankey plots. Explorative interaction analyses were performed between scores, measurements, and atrial fibrillation status. In addition, we determined the correlation of manual and automated measurements with invasive PCWP in the test cohort. Finally, we evaluated the ability of automated and manual scores to discriminate between HFpEF (*n* = 182) and non-HFpEF (*n* = 245) in the diagnostic validation cohort by comparing the areas under the curve (AUCs) of the receiver operating characteristics (ROC) curves.^17,18^ We determined 95% confidence intervals (CI) for paired AUC differences using DeLong’s method.^19^ Statistical analyses were performed using R version 4.5 (R Core Team, 2025), and two-sided *P*-values < 0.05 were considered statistically significant.

## Results

Baseline characteristics of the three cohorts are presented in *Table 1*. In the test and ambulatory validation cohorts, most patients were female, overweight, and symptomatic, and they commonly had a history of atrial fibrillation and hypertension.

**Table 1:** Baseline characteristics of the three study cohorts

Demographics	Test cohort (*n* = 102)	Ambulatory validation cohort (*n* = 129)	Diagnostic validation cohort (*n* = 427)
Age (years), mean (SD)	73.61 (8.74)	81.4 (8.55)	67.0 (11.4)
Women (%)	68 (68.7%)	93 (72.1%)	212 (49.5%)
NYHA II	39 (39.4%)	65 (50.4%)	93 (21.7%)
NYHA III	58 (58.6%)	53 (41.1%)	52 (12.1%)
Body mass index (kg/m^2^), mean (SD)	28.43 (5.71)	29.6 (6.06)	30.2 (6.9)
Medical history			
Atrial fibrillation	47 (47.5%)	83 (64.3%)	113 (26.4%)
Diabetes mellitus	31 (31.3%)	28 (21.7%)	NA
Hypertension	65 (65.7%)	61(47.3%)	271 (63.3%)
COPD	16 (16.2%)	11 (8.5%)	NA
Myocardial infarction	15 (15.2%)	14 (10.9%)	NA
NT-proBNP (pmol/L), median (IQR)	103 (54–230)	74 (39–177)	15.7 (5.5–62.4)

COPD, chronic obstructive pulmonary disease; NA, not available; NYHA, New York Heart Association; NT-proBNP, N-terminal pro-B-type natriuretic peptide.

### Manual and automated echocardiographic measurement and diagnostic scores

*Table 2* depicts the yield of manual and automated echocardiographic measurements and their correlation. Missing measurements were related to poor image quality. The proportion of cases in which the algorithm returned all echocardiographic components of both scores were 42%, 82%, and 49% in the test cohort, ambulatory validation cohort, and diagnostic validation cohort, respectively. Manual and automated echocardiographic measurements showed reasonable to high correlation coefficients ranging from 0.43 to 0.93 (*Table 2*). Similarly, manual and automated HFpEF scores showed good agreement in all cohorts with correlation coefficients ranging between 0.78 and 0.86 for the HFA-PEFF score and between 0.96 and 0.98 for the H_2_FPEF score (*Table 2*).

**Table 2:** Yield and correlation between manual and automated HFA-PEFF and H_2_FPEF scores and for selected echocardiographic parameters.

	Test cohort (*n* = 102)				Ambulatory validation cohort (*n* = 129)				Diagnostic validation cohort (*n* = 427)			
	Yield manual	Yield automated	r	RMSE	Yield manual	Yield automated	r	RMSE	Yield manual	Yield automated	r	RMSE
HFA-PEFF	NA	NA	0.78	0.79	NA	NA	0.80	0.88	NA	NA	0.86	0.98
H_2_FPEF	NA	NA	0.97	0.79	NA	NA	0.98	0.41	NA	NA	0.96	0.62
LVEF	100	74	0.53	9.67	64.3	98.4	0.59	7.03	94.9	98.8	0.53	8.80
e′ septal	99	97.9	0.84	1.44	100	100	0.93	0.77	86.9	94.2	0.04	4.67
e′ lateral	99	97.9	0.83	2.03	100	100	0.93	1.06	87.1	94.9	0.12	3.60
E/e′ mean	97.9	85.4	0.92	2.84	100	100	0.94	1.4	79.7	93.7	0.26	6.52
LVMI	94.8	70.8	0.62	27.6	100	100	0.76	13.8	97.9	96.7	0.73	21.94
RWT	97.9	74	0.43	0.15	100	100	0.46	0.1	36.7	98.6	0.13	0.41
LVPWd	99	77.1	0.46	2.18	100	100	0.60	1.99	98.8	98.6	0.51	8.43
LAVI	66.7	74	0.74	15.22	100	100	0.86	12.51	92.5	57.0	0.74	8.74
PASP	76	77.1	0.85	36.21	100	100	0.79	7.97	45.6	74.3	0.65	12.61

LVEF was measured using Simpson’s biplane method if both apical four-chamber (A4C) and apical two-chamber (A2C) views were available, or using A4C or A2C if both were not available. Manual relative wall thickness (RWT) was calculated as 2*LV posterior wall thickness/LV internal end-diastolic diameter. Manual PASP was calculated as 4*(tricuspid regurgitation peak jet velocity)^2.

e′ lateral, early diastolic tissue velocity at the lateral mitral annulus; e′ septal, early diastolic tissue velocity at the septal mitral annulus; LAVI, left atrial volume index; LVEF, left ventricular ejection fraction; LVMI, left ventricular mass index; LVPWd, left ventricular posterior wall thickness in diastole; E/e′, ratio of early mitral inflow velocity to early diastolic tissue velocity; NA, not applicable; PASP, pulmonary artery systolic pressure; RMSE, root mean squared error; RWT, relative wall thickness.

**Figure 1:**
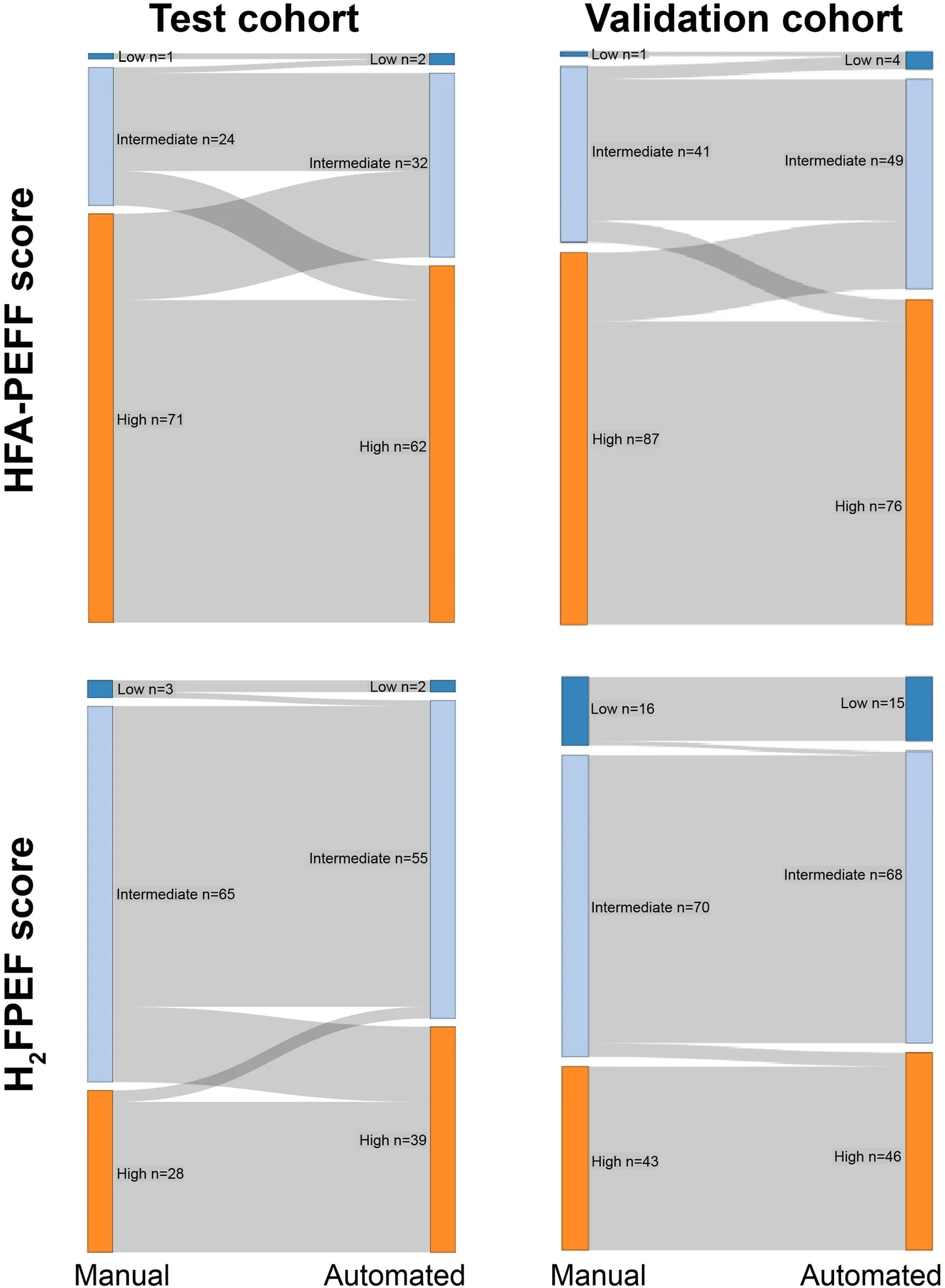
Sankey plots showing the classification of patients into a low, intermediate, or high probability of HFpEF using manual and automated HFA-PEFF score (upper panel) and the H_2_FPEF score (lower panel) in the test and ambulatory validation cohorts. HFpEF, heart failure with preserved ejection fraction.

*Figure 1* shows the re-classification of patients into low, intermediate, and high risk of HFpEF using manual and automated HFpEF scores. In the test cohort, 21% of 71 patients with high-likelihood HFpEF based on manual HFA-PEFF scores were classified as intermediate likelihood using automated HFA-PEFF scores (18.6% of 87 patients in the ambulatory validation cohort). This was mainly due to smaller left atrial volume index (LAVI) estimations by the automated vs. manual echocardiographic measurement [i.e. ambulatory validation cohort LAVI 38 (32; 49) vs. 47 (40; 60) mL/m^2^, respectively; Bland–Altman bias −9.9 (−11.3–8.6)], which did not consistently differ by atrial fibrillation status (*P*-interaction 0.63, 0.57, and <0.001, in the test, ambulatory, and diagnostic cohort, respectively).

### Association with pulmonary capillary wedge pressure

**Figure 2:**
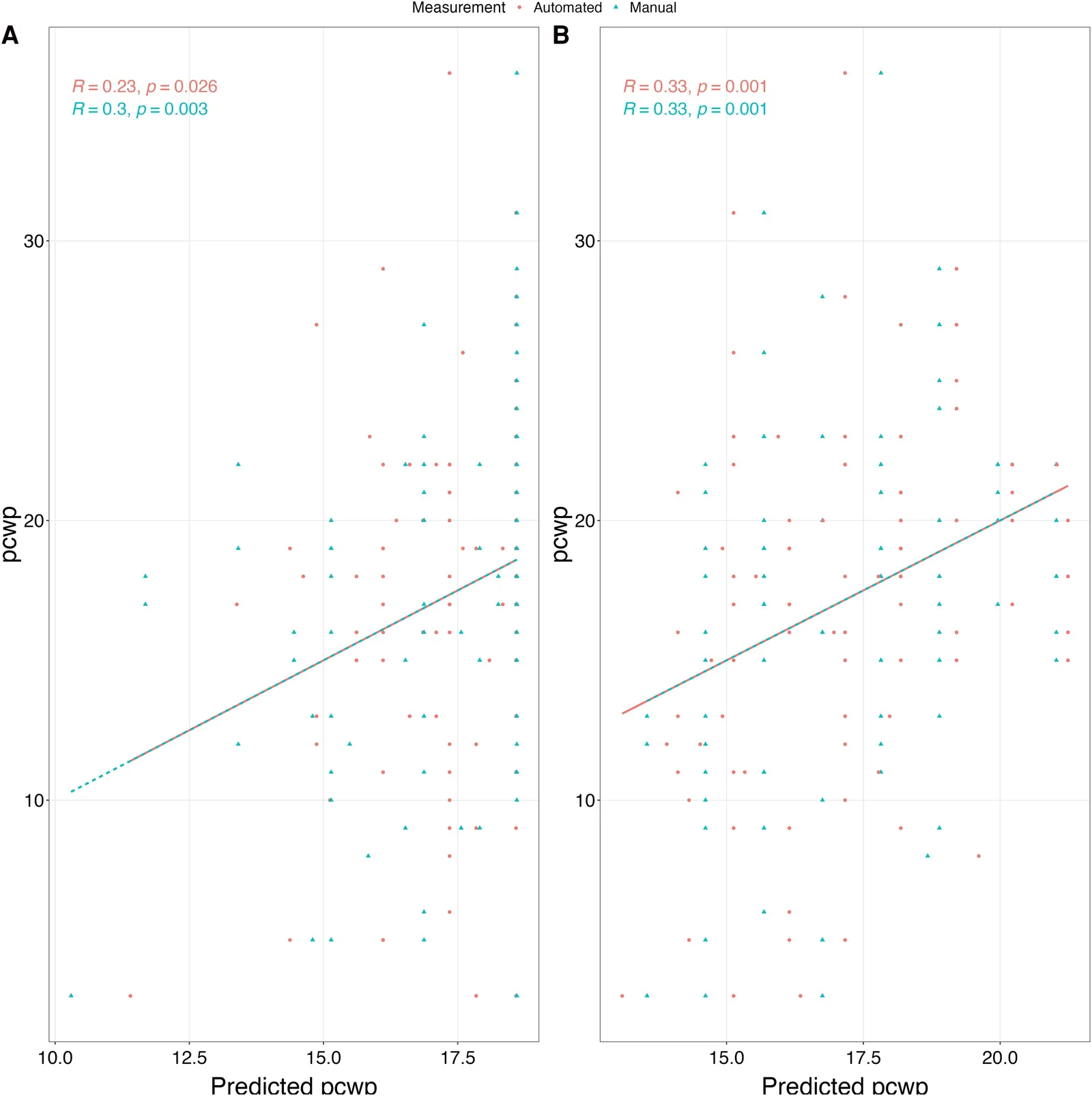
Predicted PCWP vs. actual PCWP for manual and automatically assessed variables included in the HFA-PEFF score (*A*) and the H_2_FPEF score (*B*).

*Figure 2* shows the association of manual and automated HFA-PEFF and H_2_FPEF scores with PCWP. The correlations of automated HFA-PEFF and H_2_FPEF scores with PCWP were not significantly different from manual scores [*r* = 0.23 vs. *r* = 0.30 (*P* = 0.25) and *r* = 0.33 vs. *r* = 0.33 (*P* = 0.93), respectively]. Of the individual echocardiographic measurements, both manual and automated E/e′ showed a modest correlation with PCWP [*r* = 0.24 (*P* = 0.021) and *r* = 0.33 (*P* = 0.003), respectively; Supplementary material online, *Table S1*].

### Classification of heart failure with preserved ejection fraction using manual and automated scores

**Figure 3:**
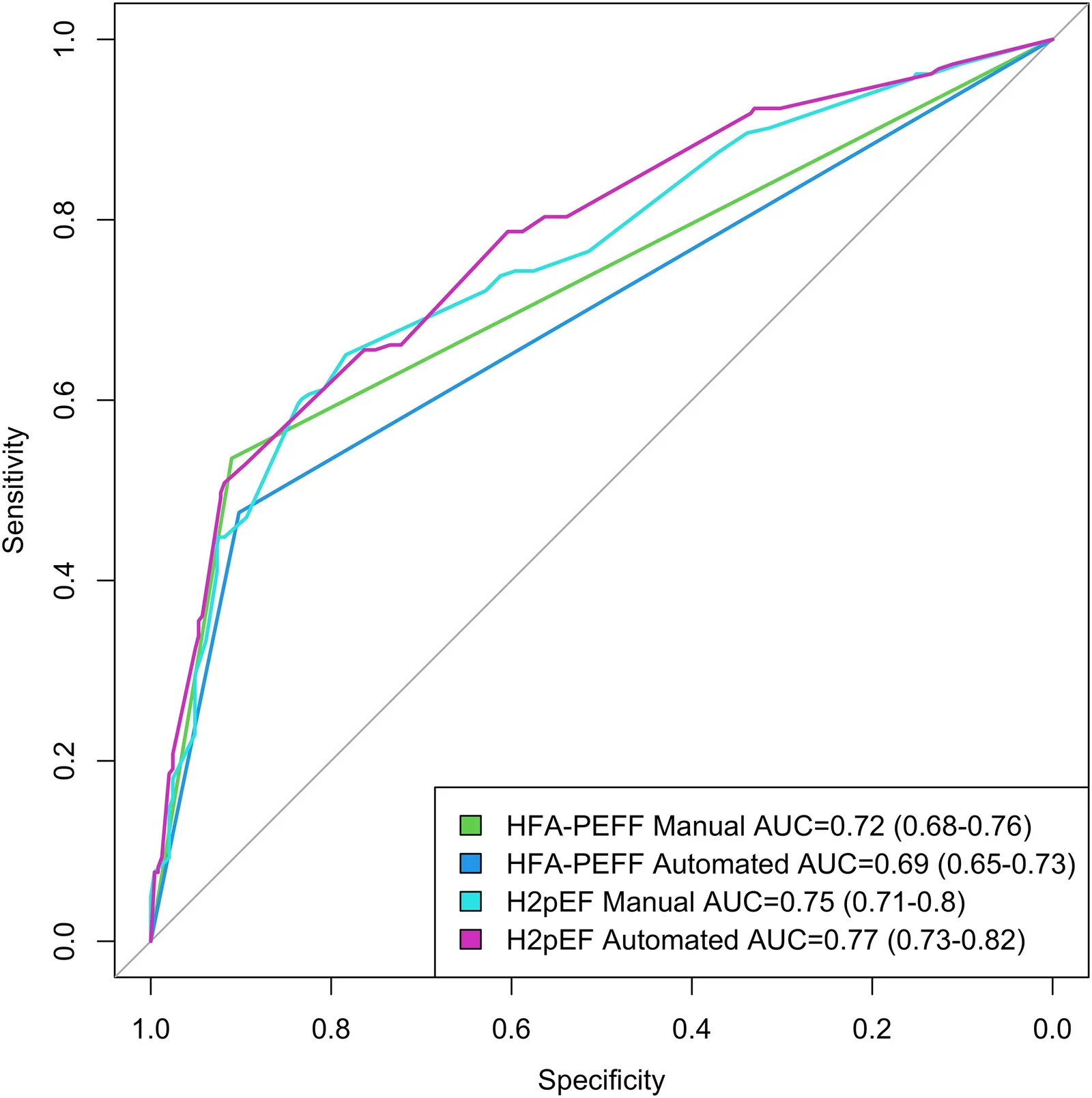
Receiver operating characteristic curves showing the ability of manual and automated HFpEF scores to discriminate between HFpEF and controls in the diagnostic validation cohort. AUCs of manual and automated HFA-PEFF scores did not differ significantly {AUC 0.72 [95% confidence interval (CI): 0.68–0.76] vs. 0.69 [95% CI: 0.65–0.73], *P* = 0.05}. The AUC of manual H_2_FPEF scores was significantly lower than automated H_2_FPEF scores [0.75 (95% CI: 0.71–0.80) vs. 0.77 (95% CI: 0.73–0.82), *P* = 0.01]. AUC, area under the curve; HFpEF, heart failure with preserved ejection fraction.

*Figure 3* depicts the ROC curves for manual and automated HFpEF scores to discriminate HFpEF from non-HFpEF in the diagnostic validation cohort. The AUCs for manual and automated HFA-PEFF were similar [0.71 (95% CI: 0.66–0.75) vs. 0.70 (95% CI: 0.66–0.74), difference 0.00 (95% CI: −0.03–0.04), *P* = 0.78], but manual H_2_FPEF scores were significantly lower than automated H_2_FPEF scores [0.75 (95% CI: 0.71–0.80) vs. 0.78 (95% CI: 0.73–0.82), difference −0.02 (95% CI: −0.04 to −0.01), *P* = 0.01]. Sensitivity, specificity, and other metrics of automated and manual HFpEF scores are provided in Supplementary material online, *Table S2*.

## Conclusions

The main findings of this study were (i) HFA-PEFF and H_2_FPEF scores determined using manual and automated echocardiographic measurements were comparable, (ii) manual and automated HFA-PEFF and H_2_FPEF scores showed similar correlations with PCWP, and (iii) diagnostic accuracy of manual and automated HFA-PEFF and H_2_FPEF scores to discriminate HFpEF from controls was similar. These findings suggest that facilitating HFpEF diagnosis using automated deep learning analysis of echocardiograms is feasible.

Diagnosing HFpEF in ambulatory patients can be challenging as dyspnoea and cardiac functional abnormalities may only manifest during exertion. Diagnostic scores were developed to assist in diagnosing patients suspected of having HFpEF. However, the practical application of these scores becomes complicated when there is disagreement between the predicted likelihoods of HFpEF.^20^ As the HFA-PEFF and H_2_FPEF scores contain different echocardiographic parameters, consistent and accurate measurements are critical to minimize disagreement. Our findings indicated that automated and manual measurements were comparable, without a consistent effect of atrial fibrillation. Manual and automated HFA-PEFF and H_2_FPEF scores showed similar but poor correlation with resting PCWP, although PCWP may be normal in resting conditions. This is in line with previous studies demonstrating that echocardiographic variables only show modest correlation with invasive hemodynamics.^21,22^ Finally, the ability of automated HFpEF scores to differentiate HFpEF was similar to that of manual scores. These findings imply that using automated echocardiographic analysis to determine HFpEF scores may facilitate and potentially accelerate the diagnostic process.

The current study underlines the promising application of artificial intelligence technologies to aid the diagnostic process. However, a limitation of this study is that echocardiograms from the cohorts were assessed according to local guidelines and not in a dedicated core lab, although this better resembles clinical practice. Nonetheless, echocardiograms from the ambulatory validation cohort that were conducted more recently compared with the other cohorts more often returned complete measurements by the algorithm. This indicates that newer and higher quality images improve performance of the automated software. Also, it should be noted that the AUC may have been inflated due to incorporation bias and inclusion of healthy controls in the non-HFpEF group. Moreover, a notable discrepancy between manual and automated measurements was observed for LA volume, leading to numerically more patients classified with intermediate instead of high risk for HFpEF according to the HFA-PEFF score. In such patients, manual reassessment of LA volume may influence diagnostic decisions and necessitate fewer additional diagnostics. Our findings, therefore, warrant prospective validation to assess whether automated HFpEF scores improve diagnostic consistency and accuracy.

In conclusion, this study demonstrated that facilitating the assessment of HFpEF scores using a fully automated deep learning echocardiography workflow yields similar results compared with manual scores. It additionally reduces the effects of interobserver variability and improves efficiency. In case of discrepancy between scores or intermediate likelihood, further investigation remains warranted, as per guideline recommendations.^3^

## Supplementary Material

ztag142_Supplementary_Data

## Data Availability

All relevant data are included in the paper, and its Supplementary material is online. The article’s data may be shared upon a reasonable request to the corresponding author.
