# Supplementary material for: Automated assessment of echocardiograms to aid diagnosis of heart failure with preserved ejection fraction
Source: Eur Heart J Digit Health. 2026 Sep 4;7(8):ztag142. doi: 10.1093/ehjdh/ztag142 (PMC13584924; doi:10.1093/ehjdh/ztag142)
Supplement: Supplementary file 1 [file ztag142_supplementary_data.docx]

# Supplementary Material

**Supplementary Table 1:** Correlation of the manual and automated HFA-PEFF and H_2_FPEF scores and echocardiographic parameters with invasive pulmonary capillary wedge pressure.

|  | **Manual** | | **Automated** | |
| --- | --- | --- | --- | --- |
|  | **Pearson r** | **P-value** | **Pearson r** | **P-value** |
| HFA-PEFF | 0.30 | 0.003 | 0.23 | 0.026 |
| H_2_FPEF | 0.33 | 0.001 | 0.33 | 0.001 |
| LVEF | 0.02 | 0.845 | -0.02 | 0.894 |
| e' septal | -0.01 | 0.959 | 0.02 | 0.831 |
| e' lateral | 0.05 | 0.608 | -0.04 | 0.679 |
| E/e' | 0.24 | 0.021 | 0.33 | 0.003 |
| LVMI | 0.18 | 0.082 | -0.04 | 0.741 |
| RWT | 0.02 | 0.833 | 0.04 | 0.728 |
| LVPWd | 0.12 | 0.259 | 0.03 | 0.794 |
| LAVI | 0.20 | 0.111 | 0.17 | 0.160 |
| PASP | 0.12 | 0.330 | -0.02 | 0.848 |
| Abbreviations: e’ lateral = early diastolic tissue velocity at the lateral mitral annulus, e’ septal = early diastolic tissue velocity at the septal mitral annulus, LAVI = Left Atrial Volume Index, LVEF = Left Ventricular Ejection Fraction, LVMI = Left Ventricular Mass Index, LVPWd = Left Ventricular Posterior Wall thickness in diastole, E/e' = ratio of early mitral inflow velocity to early diastolic tissue velocity, PASP = Pulmonary Artery Systolic Pressure, RWT = Relative Wall Thickness. | | | | |

**Supplementary Table 2:** Threshold metrics of manual and automated HFA-PEFF and H_2_FPEF scores for the diagnosis of HFpEF versus non-HFpEF in the diagnostic validation cohort. Score thresholds for classification of HFpEF were defined according to guidelines.

|  | **Accuracy** | **Sensitivity** | **Specificity** | **PPV** | **NPV** | **Prevalence** |
| --- | --- | --- | --- | --- | --- | --- |
| HFA-PEFF manual | 0.73 | 0.90 | 0.51 | 0.71 | 0.79 | 0.57 |
| HFA-PEFF automated | 0.73 | 0.91 | 0.49 | 0.71 | 0.80 | 0.57 |
| H_2_FPEF manual | 0.70 | 0.92 | 0.41 | 0.68 | 0.80 | 0.57 |
| H_2_FPEF automated | 0.74 | 0.92 | 0.49 | 0.71 | 0.82 | 0.57 |
| Abbreviations: NPV: negative predictive value; PPV: positive predictive value. | | | | | | |
